# Numerical Simulation and Experimental Confirmation of a Bimetallic Pipe Forming Process

**DOI:** 10.3390/ma13163561

**Published:** 2020-08-12

**Authors:** Zhiqiang Dong, Zhenzhen Xu, Wenke Wang, Zongyue Bi, Jianxun Zhang

**Affiliations:** 1 State Key Laboratory for Mechanical Behavior of Materials, Xi’an Jiaotong University, Xi’an 710049, China; dongzhiqiang@stu.xjtu.edu.cn (Z.D.); badfish0440@stu.xjtu.edu.cn (Z.X.); wwk2015@stu.xjtu.edu.cn (W.W.); 2 Baoji Petroleum Steel Pipe Co., Ltd., Baoji 721008, China; bsgbzy@cnpc.com.cn

**Keywords:** bimetallic pipe, JCO, stress, simulation, digital image correlation

## Abstract

Most oil and gas is transported by pipeline, and corrosion causes a great threat to the service life of the pipeline; bimetallic pipe, which combines the advantages of good mechanical properties, good corrosion resistance, and relatively low price, is a good choice for high-pressure and corrosion-resistant pipe, but its manufacturing process and stress distribution are more complex than single metal pipe. JCO is a widely used cold forming method for pipes which is named by the shape of the plate in the forming process, i.e. J-shape, C-shape and O-shape, and the forming process is an important parameter that determines the level of imperfections and residual stresses in a pipe, and residual tensile stress will accelerate corrosion failure of the pipe. In this study, the three-dimensional (3D) finite element method (FEM) is used to simulate the pre-bending and JCO forming process of a 2205/X65 bimetallic pipe. The model and the simulated results are validated by digital image correlation (DIC) experimental and the opening width of the formed pipe billet, respectively. The influence factors of the stresses are studied. Further, a two-dimensional (2D) model is established to study the characteristics of bimetallic plate bending and the stress distribution at the interface of different materials, and the results are compared with that of three-dimensional model.

## 1. Introduction

Piping systems play a critical role in the transportation of chemicals, both liquids and gases, from one location to another. The residual stress produced in the process of pipe manufacturing accelerates the corrosion, fatigue, and creep failure of pipes [1,2], which cause enormous economic losses. Bimetallic pipes, in which two materials are metallurgically bonded, combine the advantages of corrosion resistance and economic viability, so that they are widely used in petroleum, nuclear power, offshore platforms, and pharmaceutical fields [3]. In these mechanically bonded pipes, under large bending loads, the liners undergo plastic deformation, which results in non-reversible liner wrinkling. This wrinkling impedes the flow and significantly reduces the performance of the pipeline [4]. Pipes are classified into ERW, JCO, and UOE pipes according to their cold forming processes. JCO forming is one of the common methods of manufacturing welded pipes. The JCO pipe manufacturing processes are composed of pre-bending forming, JCO forming, welding, and expansion stages. During the pre-bending forming stage, the plate edges are bent into circular arcs over a width of approximately one radius on each side. Subsequently, the plate is formed into a J-shape, then pressed into a C-shape by progressive a multi-step air bending, and, finally, is formed into an O-shape. Therefore, the JCO forming quality depends on the deformation of the air bending. The JCO process is adaptable, economical, and suitable for medium-scale production. It has been widely used in the Panyu–Huizhou pipeline, Shaanxi–Beijing pipeline, Zhongwu pipeline, West–East Gas Pipeline Project, and other land pipelines of China [5,6,7,8].

ERW pipes are produced from roll forming, which is an economical and highly productive method in plate forming technology. The finite element (FE) method has been widely used in the design and implementation of metal forming to predict the distribution of the stress and strain in the formed part [9,10]. Kim et al. [11] established a rigid–plastic FE model to predict the edge shape of the initial strip for a thick tube roll forming. Jiang et al. [12] simulated the entire rolling forming process of a cage using an explicit elastic–plastic FE model, and studied the strip deformation. The UOE process is the most effective method for manufacturing large-diameter, thick-wall, and high-strength longitudinally submerged arc welded pipes. Ren et al. [13] established a two-dimensional (2D) FE model of the UOE forming process. The effects of the process parameters, friction coefficient, and material properties on the slotting and ellipticity were studied numerically.

There are few reports on the application of a three-dimensional (3D) model in the simulation of large-scale pipeline forming processes. Gao et al. [14] and Luo et al. [15] established 2D FE models to study the JCO forming process of a pipe and studied the stress distribution and appropriate punch displacement. It is extremely difficult to measure the residual stress of large-diameter pipeline, and the evolution law of stress is not fully revealed. Ren et al. [16] measured the residual stress in a pipeline via the neutron diffraction technology. They stated that, for such large components, residual stress measurement required tremendous preparation and planning. Chen et al. [17] studied the effect of the type I residual stress on the pitting corrosion and stress corrosion crack formation of pipeline steel. The results showed that the tensile residual stress was a large mechanical driving force for the crack nucleation and short crack propagation, adversely affecting the safety and service life of pipeline steel. Owing to the springback in a sheet metal, the bending process is affected by numerous factors; hence, it is difficult to accurately predict the shape of a tube after the JCO forming. Hino et al. [18] studied the springback in the draw-bending process of a two-layer plate. The experimental and analytical results exhibited that the shape of the tube after the JCO forming was related to the springback in the plate bending process. The springback of plate laminates is significantly influenced by the strength difference between the layers, relative positions of the weak/strong layers, annual thickness ratio, and tensile force acting on the laminates. Ling et al. [19] studied the effect of die parameters on the springback. Therefore, it is necessary to discuss the stress and shape of a pipe after forming by numerical calculation. In addition, Gao et al. [20] stated that the FE method was helpful in rapidly obtaining the appropriate JCO forming process parameters in the development and design stages of new pipelines. It subsequently allowed to improve the quality of the work piece, shorten the designing period, and cut down the cost of the pre-production testing. In an actual production, the stress and deformation of a billet in the forming process will be transferred to the next process. First, the size of the forming stress will affect the quality of the subsequent welding process, and second, far from the welding position, the distribution of the stress in the pipeline is mainly caused by the forming process. Pipelines are frequently used to transport corrosive oil and gas, and they endure large working stresses during transportation. The superposition of the residual stress and working stress in the production process may cause a local deformation of the pipeline, which will affect its use. In addition, stress corrosion is one of the main forms of pipeline damage. Therefore, when the residual tensile stress is high, it will affect the bearing capacity of the pipeline, accelerate the corrosion damage of the pipeline, and ultimately affect the service life of the pipeline. Using the FE method to study a pipe forming process, the evolution of the stress throughout the process and the distribution of the residual stress at each location of the pipe will be known clearly. These will provide guidance for developing comparatively better stress distributions and smaller tensile residual stress processes.

FEM of the JCO forming process of a pipe is a multi-non-linear (material, geometric, and contact non-linear), elastic–plastic and, large-deformation problem, and few studies have been conducted on the bending of a bimetallic plate. X65 pipe steel (X65) offers high strength, high toughness, and a minor Bauschinger effect; 2205 duplex stainless steel (2205) is particularly suitable for the production of the stainless steel bimetallic plate for use in oil and gas transportation pipes and refining equipment; 2205/X65 bimetallic plate is fabricated by the explosive welding technique, the interface of explosively welded 2205/X65 bimetallic sheet exhibited reliable and robust shear strength and bending properties, and a metallurgical combination is formed between 2205 and X65, which has been investigated by Zhang et al. [21]. Gou et al. [22] studied the butt-welding process of a 2205/X65 bimetallic plate. Dong and Zhang [23] investigated circumferential welds made of a 2205/X65 bimetallic pipe. In this study, using ABAQUS commercial FE calculation software, the pre-bending and JCO forming FE models of a 2205/X65 bimetallic pipe were established. The FE simulation results were compared to the results measured by digital image correlation (DIC) techniques to validate the model. The stress distribution and shape of the 2205/X65 bimetallic pipe after the pre-bending and JCO forming were obtained by calculation. Furthermore, the stress evolution in the process of the JCO forming and the effect of the material strength, plate sizes, and forming parameters on the stresses were studied. The interfacial stress distribution of different materials was analyzed by a 2D model with fine grids, and the simulated results of the 2D and 3D models were compared. Moreover, the free bending characteristics of bimetallic plates were highlighted.

## 2. Materials and Methods

**Figure 1:**
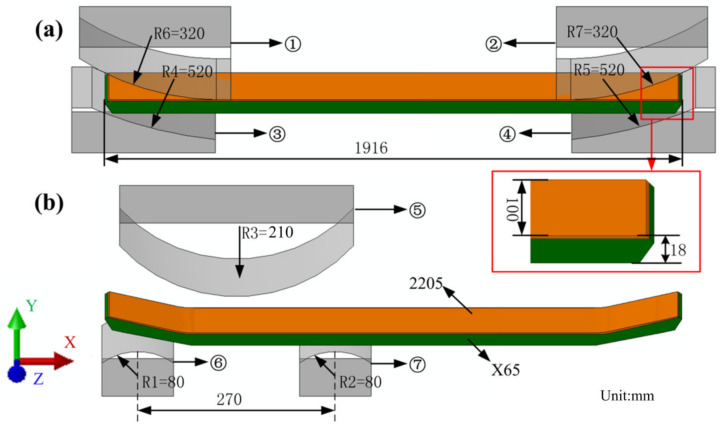
Three-dimensional finite element (FE) model for: (**a**) pre-bending and (**b**) the JCO forming process.(① and ② are the upper pre-bending dies, ③ and ④ are the lower pre-bending dies, ⑤ is the upper JCO forming die, and ⑥ and ⑦ are the lower JCO forming dies).

**Figure 2:**
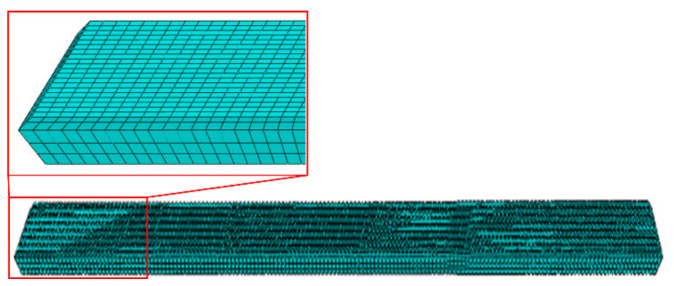
Mesh division of the plate in the three-dimensional (3D) model.

**Figure 3:**
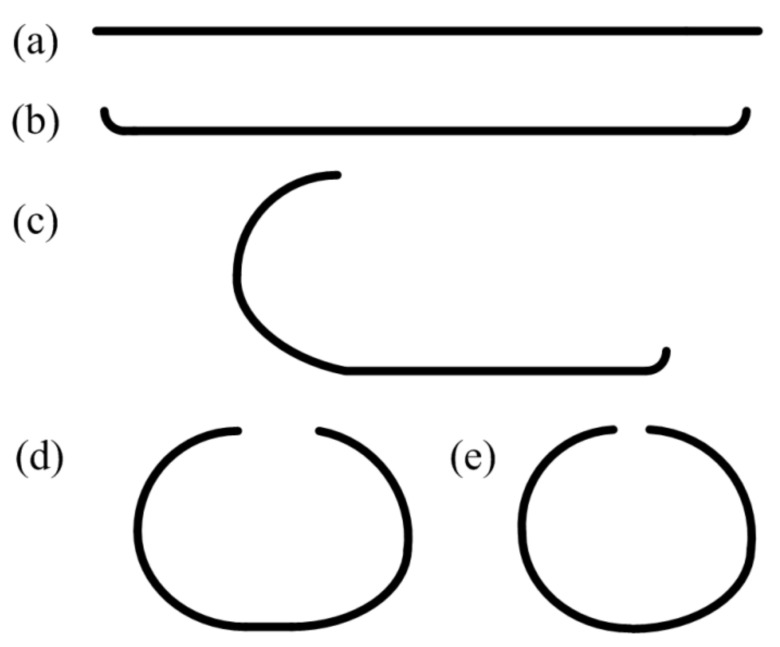
Schematic of JCO pipe forming process: (**a**) initial plate; (**b**) pre-bending; (**c**) J-forming; (**d**) C-forming; (**e**) O-forming.

**Figure 4:**
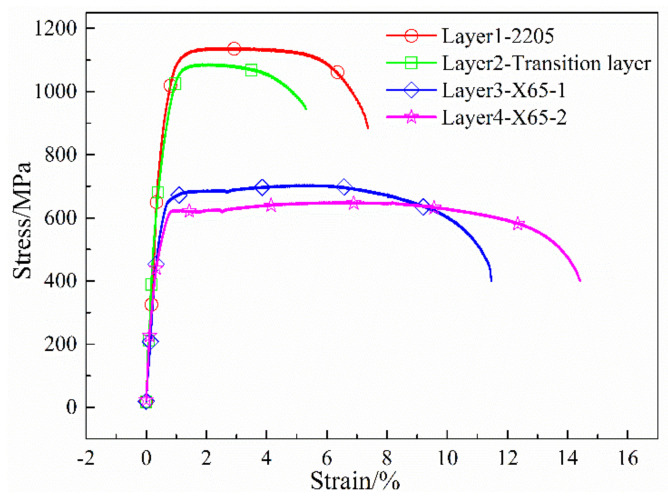
Stress-strain curves of materials for 2205/X65 plate [21].

The 3D FE model of a 2205/X65 bimetallic pipe undergoing pre-bending and the JCO forming process is established by ABAQUS software, as shown in Figure 1. The size of the bimetallic plate is 1916 mm × 100 mm × 18 mm, and the length of the circumference of a Φ610 mm pipe is 1916 mm. The thickness of 18 mm is composed of two parts: the upper layer is 2205 stainless steel with a thickness of 2 mm, and the lower layer is X65 steel with a thickness of 16 mm. Grooves at both ends of the plate are formed for the subsequent welding process. Apart from this plate, there are four pre-bending dies (in the upper part of Figure 1) and three JCO forming dies, in which specific size parameters are shown in Figure 1. The span is 270 mm, which is the distance between the two lower JCO forming dies, and the press times are 17. All the dies are modeled as analytical rigid bodies. The contact between a die and the plate is set as a “master–slave” surface-to-surface condition, where the die is the master contact surface and the corresponding surface of the bimetallic plate is set as the slave surface. The contact property is the penalty function friction, and the friction coefficient is 0.1. The coordinate system used in this study is shown in Figure 1, where the X, Y, and Z axes coincide with the length, thickness, and width direction of the plate, separately. The plate is discretized by an eight-node linear brick hourglass control element, C3D8R. The maximum and minimum element sizes are 5.3 mm × 5 mm × 5 mm and 2 mm × 5 mm × 5 mm, respectively, as shown in Figure 2. The ABAQUS/standard solver was employed. The calculating process could be described as follows: First, the plate was lifted up for pre-bending and then lowered down to press one side of the plate into a J-shape once the pre-bending process was finished. Following, the other side of the plate was pressed into a J-shape and then into a C-shape; finally, the middle of the plate was pressed to form an open O-shape. The Schematic of JCO pipe forming process is shown in Figure 3. The chemical composition of X65 pipe steel and 2205 duplex stainless steel are shown in Table 1. The densities of the X65 pipeline steel and the 2205 duplex steels are 7.85 × 10^−9^ tonne/mm^3^ and 7.80 × 10^−9^ tonne/mm^3^, respectively. Both have a Young’s modulus of 210 GPa and Poisson’s ratio is 0.3, as shown in Table 2. Before the explosive welding, the 2205 plate had a tensile strength of 755 MPa, the same for the X65 plate was 568 MPa [21]. Tsai and Chen [24] and Fukuda et al. [25] obtained similar tensile results for X65 and 2205. After the explosive welding, the tensile strength of the 2205 layer increased to 1137 MPa, and that of the X65 layer, which was far from the transition layer, increased to 630 MPa. These were due to the severe deformation and working-hardening during the explosive welding of the bimetallic plate. Elastic-plastic model following Von Mises yield criterion with an isotropic hardening law is adopted for the simulation of the material behavior of the pipe. Isotropic hardening model is useful for cases where the straining at each point is essentially in the same direction throughout the analysis. The stress-strain curves used in this simulation are shown in Figure 4. In the 3D model, the material properties of the 2205/X65 bimetallic plate are simplified to the 2205 layer with a thickness of 2 mm and the X65-2 layer with a thickness of 16 mm.

## 3. Results and Discussion

### 3.1. Confirmation of Finite Element Model

**Figure 5:**
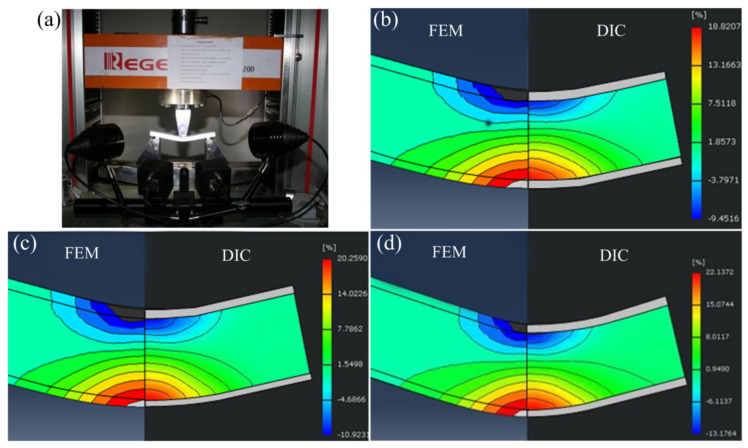
Digital image correlation (DIC) test and the results compared with the FE simulation results: (**a**) DIC testing equipment and process, (**b**) DIC and finite element method (FEM) results with 20-mm press amount, (**c**) DIC and FEM results with 21-mm press amount, and (**d**) DIC and FEM results with 22-mm press amount.

To validate the established FE model of the sheet metal bending, an FE model with the same material composition, material properties, and FE settings as those mentioned in Section 2 was established. These were in addition to its length of 210 mm, width of 20 mm, and span of 164 mm. Based on a three-point bending experiment, the bending process of the explosive composite plate was tested using an XJTUDIC 3D digital speckle dynamic strain measurement and analysis system. The strain test equipment is displayed in Figure 5a. The XJTUDIC 3D digital speckle dynamic strain measurement and analysis system is based on the 3D digital speckle correlation method. In combination with the binocular stereo vision technology, two high-speed cameras are used to capture the speckle images in real-time at each deformation stage of an object. The digital speckle correlation algorithm is continuously improved to match the deformed points on the surface of the object. Three-dimensional coordinates of the matching points are constructed. Finally, the displacement field data are computed and processed, and the deformation information is visualized. Nixon et al. [26] had measured the strain of a high-purity titanium material in a four-point bending test by the DIC method, and compared it with the results of the FE method. In this study, the plate size, material composition, forming parameters, and FE model used in the experiment are the same. The experimental results of the DIC algorithm are compared to those of the FE method. The horizontal component of the true strain is extracted when the plate is depressed to the lowest point, and the cloud image is divided into the same regions. The strain range is adjusted to the same extent, and a contour map is drawn. Finally, the experimental results of the DIC and simulation results of FEM with press amounts of 20 mm, 21 mm, and 22 mm are compared. The comparison of the axial strain results is shown in Figure 5b–d. From the figure, it can be seen that the results of the DIC test and FE simulation are in good agreement, which exhibits the validity and reliability of the established FE model. Table 3 lists the spring-back and bending angle of the plate after unloading as obtained by the DIC and FEM. From Table 1, it can be seen that the relative errors between all the results of the DIC experiment and FE simulations are within 5%, which again verifies the validity and reliability of the FE model.

### 3.2. Pre-Bending

**Figure 6:**
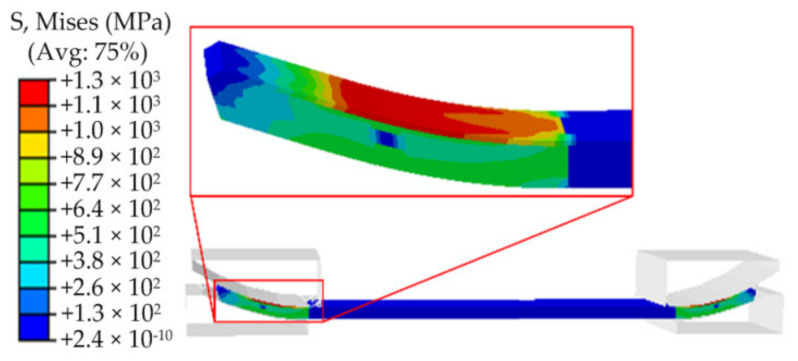
The Mises stress contour of the pre-bending after loading.

**Figure 7:**
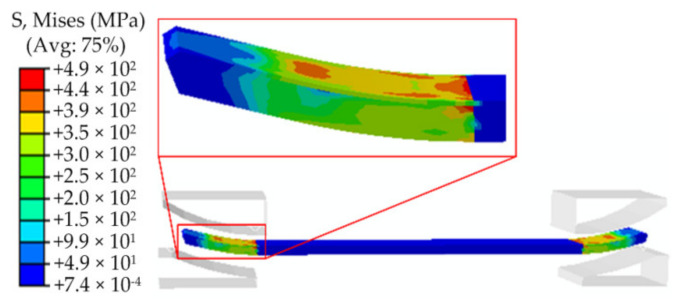
The Mises stress contour of the pre-bending after unloading.

Pre-bending is the first bending process of the JCO forming of the pipe. The result of pre-bending directly affects the following welding process and the quality of the pipe. The calculation steps can be described as follows: first, the plate is moved up to the pre-bending die, and then the lower die (punch) is moved upward while the upper die is kept stationary until the plate is pressed against the die. This is followed by unloading, then the plate is moved to the JCO forming die to complete the JCO forming of the pipe. The pre-bending simulated results are shown in Figure 6 and Figure 7. Figure 6 is the Mises stress contour of the pre-bending after loading, where the maximum Mises stress reaches to 1300 MPa, which exceeds the yield strength of the plate. The maximum stress appears on the upper surface of the plate. After unloading, the plate shows a certain degree of springback deformation. The maximum Mises stress drops to 490 MPa, which is located at the upper surface of the plate, as shown in Figure 7.

### 3.3. JCO Forming

**Figure 8:**
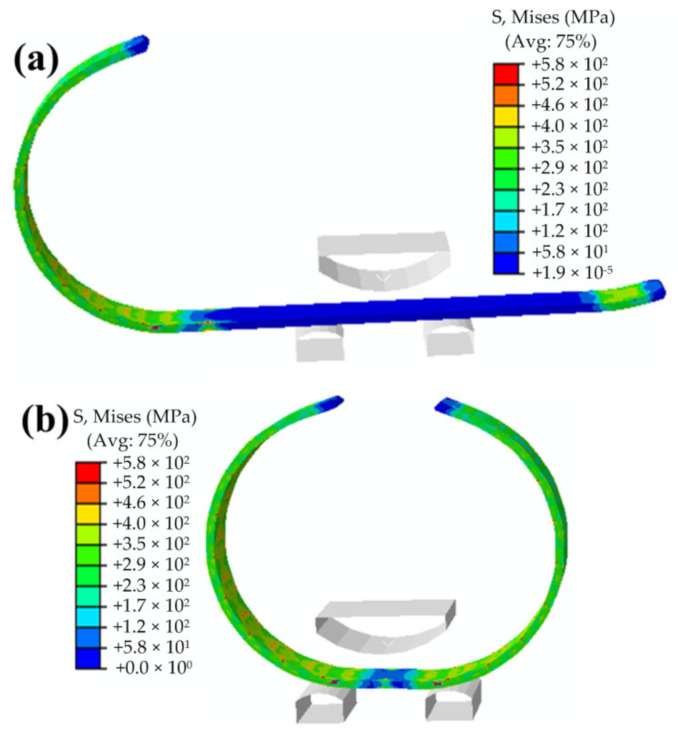
Mises stress contours: (**a**) “J” forming and (**b**) “C” forming.

**Figure 9:**
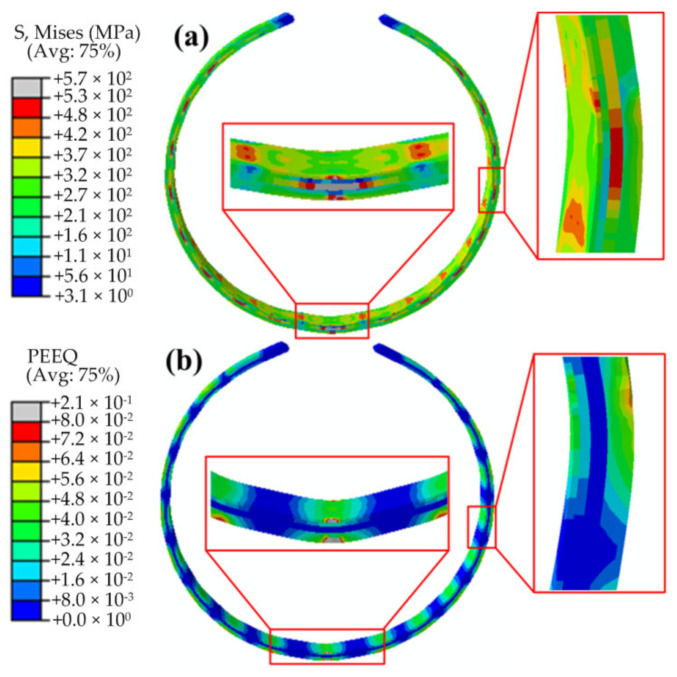
Mises stress and equivalent plastic strain (PEEQ) contours of the “O” forming: (**a**) Mises stress and (**b**) PEEQ.

Figure 8a and bare the Mises stress contours after the “J” forming and “C” forming, respectively. Figure 8a,b are the stress and equivalent plastic strain (PEEQ) contours after the “O” forming, respectively. From these figures, the stress distribution and PEEQ distributions of the pipe during the forming process can be visually seen. From Figure 8, it can be seen that the stress and equivalent plastic strain on the pipe present segmented distributions. The values and distributions of the stress and PEEQ of each segment are basically the same, except for the middle part of the plate. The last step of the pipe forming is the “O” forming. The pressing of the middle part is a symmetrical bending forming, whereas the other steps are asymmetrical bending forming. Hence, the stress and deformation in the middle of the plate are different from those in the other parts. The stress and PEEQ in the middle part of the plate, i.e., the pressing position of the “O” forming, are larger than those in the other parts. Moreover, the number of segments is consistent with the pressing time. From Figure 9a, it can be observed that a large residual Mises stress is distributed on the inner surface and in thickness direction of the pipe. It can be noted from Figure 8b that each segment has a region of zero PEEQ, indicating that the formed billet is composed of circular and straight sections, which are separated. Further, there is also a zero PEEQ part in the thickness direction. This is the neutral layer of the plate bending forming, which is located close to the 2205 side, and not in the middle of the thickness direction. This is also the difference between bimetallic pipe bending forming and single metal pipe bending forming.

**Figure 10:**
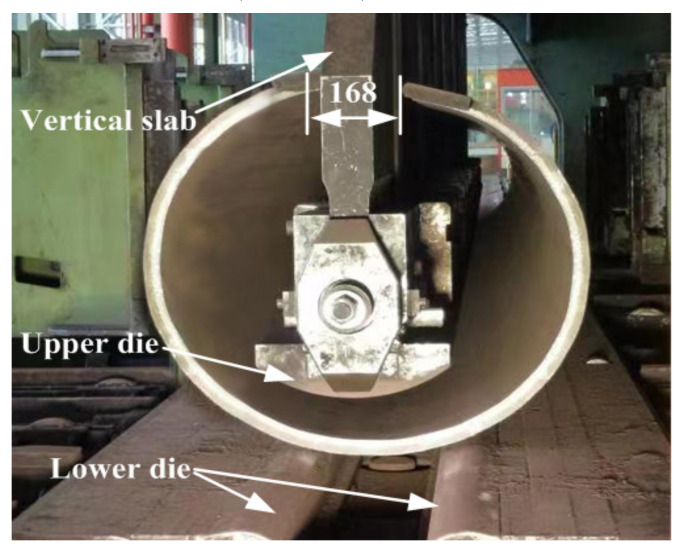
Pipe billet after “O” forming.

**Figure 11:**
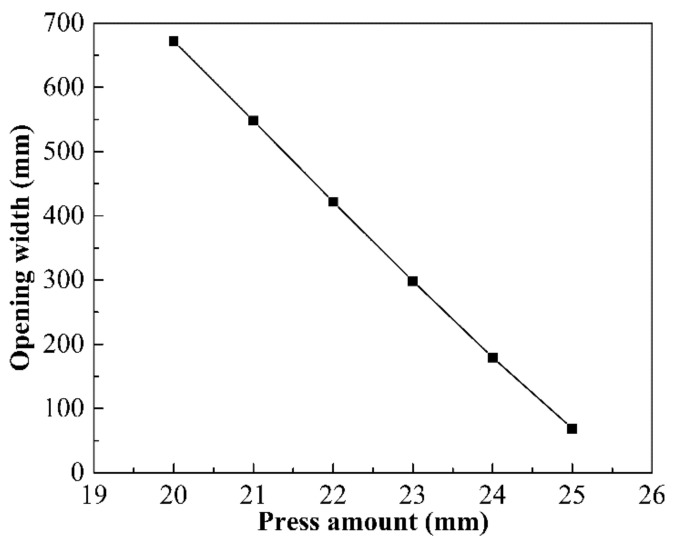
Effect of the press amount on the opening width.

Because of the restriction of the vertical slab on the top of the JCO upper die, as shown in Figure 10, the opening width of the pipe billet should be more than 110 mm. To ensure the following welding quality and reduce the residual stress, the opening width of the pipe after forming should be less than 260 mm. The press amount of the upper die is one of the key parameters determining the opening width of the pipe billet. To obtain the ideal opening width, press amounts of 20 mm, 21 mm, 22 mm, 23 mm, 24 mm, and 25 mm are studied. It can be seen from Figure 11 that, when the other factors are unchanged, the opening width decreases linearly with the increase in the press amount. It also can be noted from Figure 10 that the opening width of the pipe billet is 180 mm when the press amount of the JCO upper die is 24 mm, which meets the requirements of pipe forming. Therefore, the appropriate press amount of the JCO upper die is 24 mm. Based on the process parameters simulated by the FE method, a 2205/X65 bimetallic pipe of the same size was manufactured by the JCO forming process, as shown in Figure 10. The opening width is an important technical index for the quality control of the pipe forming, which reflects the deformation of the pipe, which in turn reflects the stress to a certain extent. Therefore, the experimental and simulation results of the opening width are compared. The opening width of the manufactured pipe is 168 mm, which is slightly smaller than the simulation result; however, it meets the requirements of pipe forming.

### 3.4. Analysis of Stress Evolution and Distribution Characteristics of JCO Pipe

**Figure 12:**
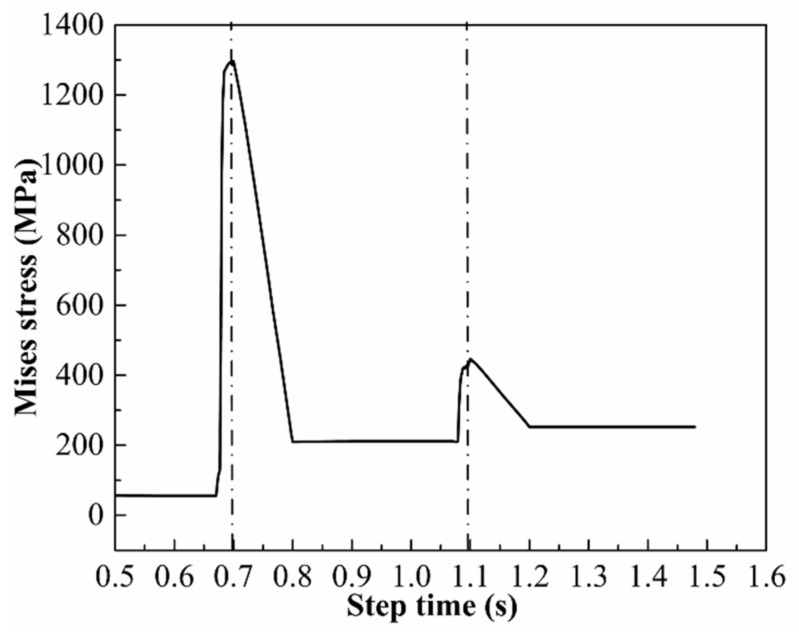
Variation in the curve of the Mises stress with the step time.

To study the evolution of the plate stress during the JCO forming, a node was selected, which was located at the center of the upper surface of the plate at the first pressing position of the die. As shown in Figure 12, 0.6–0.7 s is the first downward pressing time, and the stress at this node increases rapidly with the downward pressing of the die. The upward time of the die is 0.7–0.8 s, and it can be observed from the figure that the elastic recovery of the plate occurs with the upward pressing of the die, and the stress decreases from 1300 MPa to 210 MPa. The second downward pressing time of the die is 1.0–1.1 s, and it can be noted from the figure that the maximum Mises stress of the plate increases from 210 MPa to 406 MPa during this time. The Mises stress at this node changes by 42 MPa after the first and second upward pressings of the die, and the subsequent upward–downward pressing processes of the die and the movement of the plate do not affect the stress at this node.

**Figure 13:**
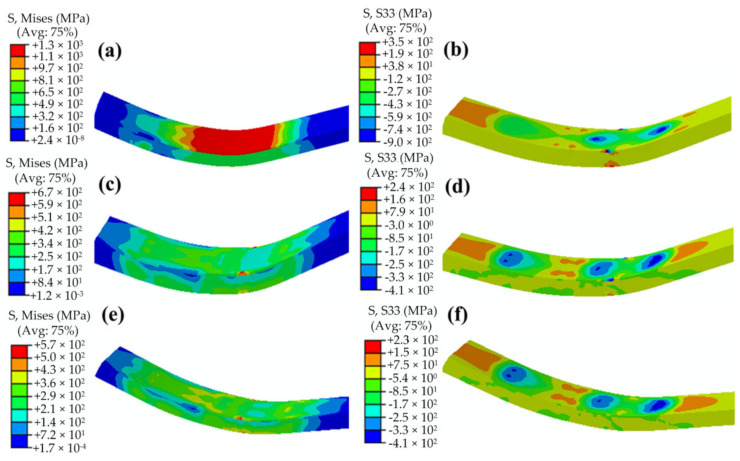
Mises and axial stress contours of the process of the JCO forming: (**a**) Mises stress contour after loading, (**b**) axialstress contour after loading, (**c**) Mises stress contour after the die upward pressing, (**d**) axialstress contour after the die upward pressing, (**e**) Mises stress contour after the plate moves forward, and (**f**) axial stress contour after the plate moves forward.

**Figure 14:**
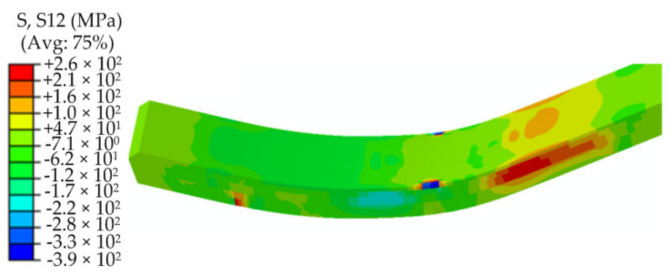
Maximum shear stress contours in the JCO forming process.

From the above research results, it can be understood that the stress distributions of the plate after the forming are segmented, and the values and distributions of the stresses in each segment are basically the same, which are mainly determined by a single downward pressing and upward of the die. Therefore, a downward–upward pressing process of the die can be selected to study the stress evolution of the JCO forming process of the plate. If a large tensile stress that is perpendicular to the thickness of the plate distribution is present on the inner surface, then the service life of the pipe will be seriously affected. The axial stress is along the width direction of the plate, which is the only large residual tensile stress in the inner surface that is perpendicular to the thickness of the plate; therefore, it is chosen as a representative stress to study the effects of the pipe size and forming parameters on the stresses after forming Figure 13 is exhibiting the evolution of the stress distributions of the plate after loading, unloading, and moving the plate forward. The Mises stress distribution after loading in the JCO forming process is the same as in the pre-bending process, and the stress on the upper surface is much larger than that on the lower surface, as shown in Figure 13a,b. It can be noted from Figure 13c,d that the plate exhibits remarkable elastic-recovery deformations and that the maximum Mises stress decreases to 670 MPa with the die upward pressing. The maximum Mises stress distributes on the upper surface edge of the 2205 stainless steel plate. In addition, the plate near the bottom of the X65 steel layer also has a large residual stress in the thickness direction. The axial stress distribution is complex, and there are compressive stresses and tensile stresses on the upper surface, with the maximum axial tensile stress of 240 MPa. It can be seen from Figure 13e,f that the distributions of the Mises stress and axial stress change little with the plate moving forward, and their values decrease slightly compared to that in the die upward stage. By studying the maximum shear stress in the forming process and comparing it with the tested shear strength, the bonding behavior of the bimetallic plate in the JCO forming process can be understood. The maximum shear stress in the JCO forming process of the pipe is shown in Figure 14. The maximum shear stress is 250 MPa, which is located in the thickness direction close to the 2205 layer. According to Zhang et al. [21], the average shear fracture strength of a bimetallic plate after explosive welding is 400 MPa. The maximum shear stress of the pipe in the JCO forming process is much less than the tested shear strength of the plate. The plate is still well bonded after the JCO forming process and has no interface separation.

### 3.5. Influences of Plate Strength, Plate Thickness, and Span on Stresses after JCO Forming of Bimetallic Pipe

This part mainly discusses the influences of the plate strength, thickness, and span on the stresses after the pipe forming, each parameter is changed within a practically acceptable range while keeping all the other parameters unchanged. The changes in the maximum hoop stress and maximum axial tensile stress on the upper surface of the plate during the downward–upward pressing process of the die and of the plate moving forward process are studied.

**Figure 15:**
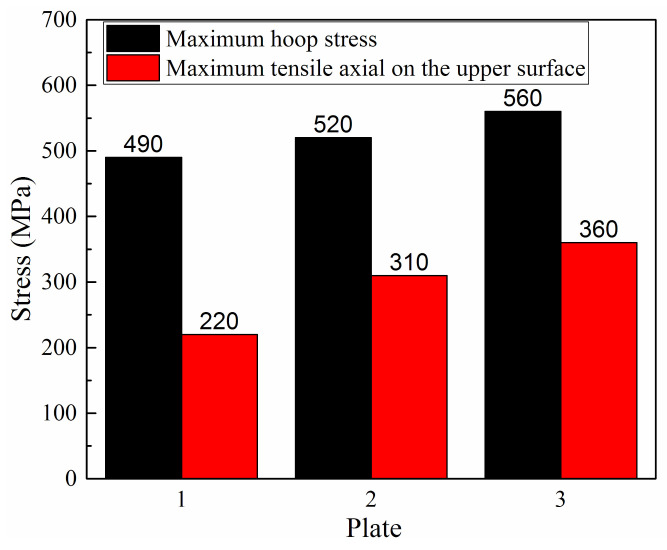
Effect of the plate strength on the residual stress.

Compared with the normal 2205 and X65 metals, the strength of the plate in this study is significantly improved, and the strength of the same batch of plate should also be different. To study the influence of the strength change of the plate on the stress during the pipe forming, three types of plates with different strengths were selected to study. Plate 1 had the strength of the X65 and 2205 plates before the explosive welding, plate 3 had the strength of the X65 and 2205 plate after the explosive welding, and plate 2 had the average strength of the plate before and after the explosive welding, which is summarized in Table 4. The results are shown in Figure 15. It can be observed from Figure 15 the maximum hoop stress and the maximum tensile axial stress on the upper surface increase with increasing plate strength. Comparing plate 3 with plate 1, the maximum hoop stress increases by 70 MPa and the maximum tensile axial stress increases by 140 MPa. Therefore, the change in the plate strength has a large influence on the stress level of the formed pipe.

**Figure 16:**
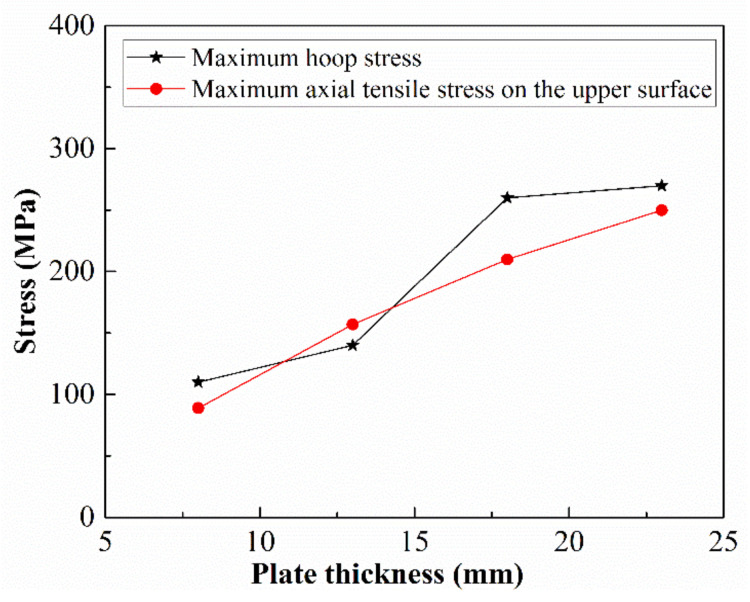
Effect of the plate thickness on the residual stress.

Four thickness sizes of 8 mm, 13 mm, 18 mm, and 23 mm were selected to study their influence on the stress of the bimetallic pipe after the JCO forming. The 2205 stainless steel layer maintained a thickness of 2 mm. As shown in Figure 16, the plate thickness has a significant influence on the maximum hoop stress and the maximum axial tensile stress on the upper surface of the plate after the forming. With the increase in the plate thickness from 8 mm to 23 mm, the maximum hoop stress increases from 110 MPa to 270 MPa, and the maximum axial tensile stress on the upper surface of the plate increases from 89 MPa to 250 MPa. It can be concluded from the above results that, with the increase in the plate thickness, the maximum tensile axial stress on the upper surface after the JCO forming increases linearly, whereas the hoop stress increases first and then tends to be stable.

**Figure 17:**
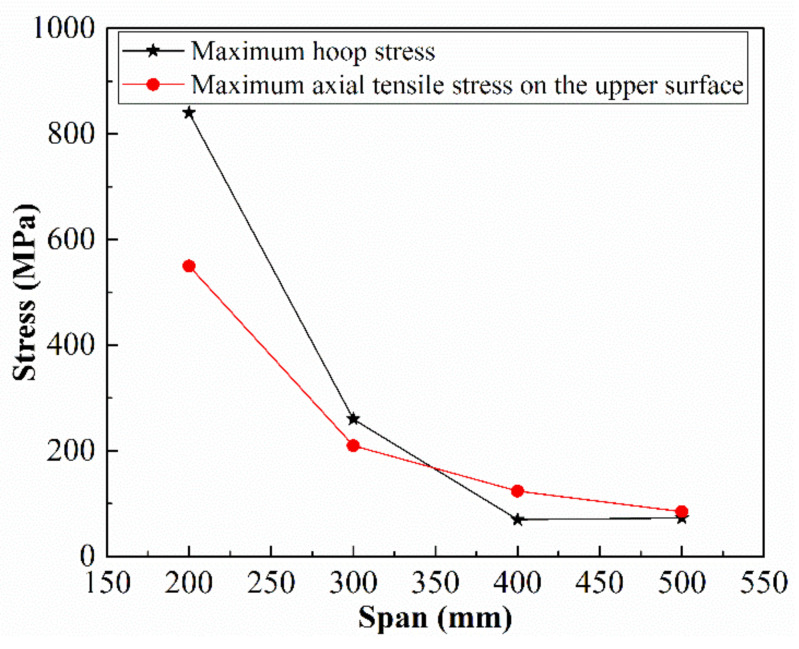
Effect of the span to the residual stress.

Finally, the influence of the span on the stress of the bimetallic pipe after the forming was studied. Four spans: 200 mm, 300 mm, 400 mm, and 500 mm, were selected for this. With the increase in the span, the maximum hoop stress and the maximum tensile stress axial on the upper surface of the plate decrease significantly, as shown in Figure 17. With the increase in the span from 200 mm to 500 mm, the hoop stress decreases from 870 MPa to 340 MPa and the maximum axial tensile stress decreases from 550 MPa to 85 MPa. When the span is 200 mm, the plate deforms severely. The results show that, with the increase in the span, the maximum tensile axial stress on the upper surface of the plate reduces significantly, whereas the hoop stress decreases first and then remains unchanged.

### 3.6. 2D Modeling and Calculated Results of JCO Pipe

**Figure 18:**
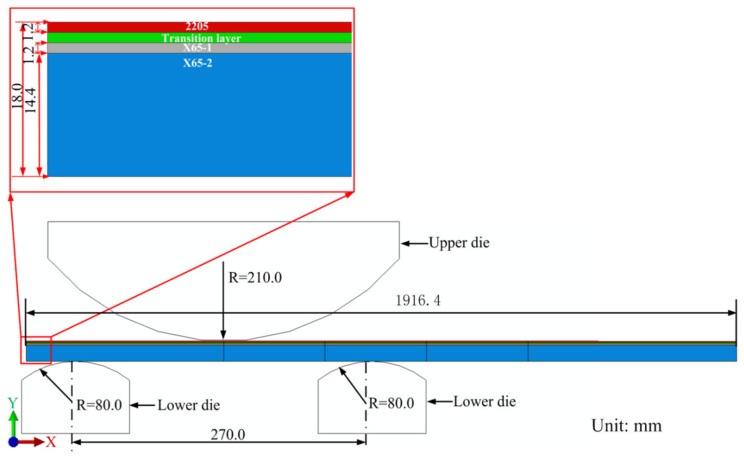
Two-dimensional (2D) finite element model for the JCO forming.

**Figure 19:**
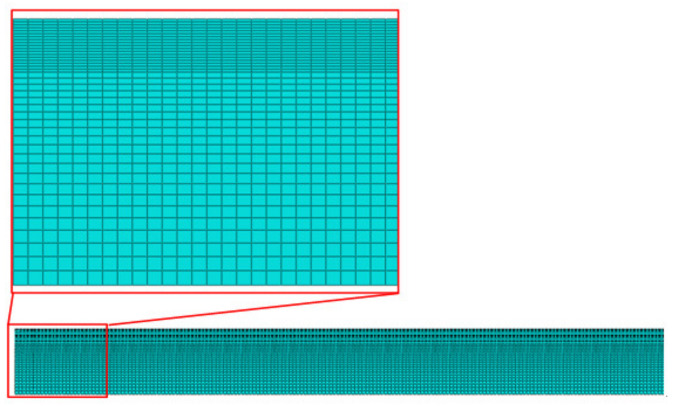
Mesh of the plate in the 2D model.

A 3D simulation is time-consuming and difficult to converge. If a 2D model could be used instead of a 3D model to study pipe pre-bending and the JCO forming process, the simulation time can be significantly reduced and the convergence of the computation will be improved Figure 18 depicts the 2D FE model for the JCO forming. The divided mesh of the plate is shown in Figure 19. The maximum element size is 1 mm × 1 mm, the minimum element size is 0.2 mm × 1 mm, and a total of 76,480 elements are divided. The element type is CPS4R, which is a four-node plane strain reduced integration quadrilateral element in ABAQUS. According to the delamination tensile test results of the 2205/X65 explosive welded bimetallic plate, the 2205/X65 plate is divided into four layers: 2205, transition layer, X65-1, and X65-2, from the upper surface to the lower surface. The thickness of the first three layers is 1.2 mm and of the fourth layer is 14.4 mm. The material properties of each layer are displayed in Figure 2. The other settings of the 2D model are the same as those of the 3D model.

**Figure 20:**
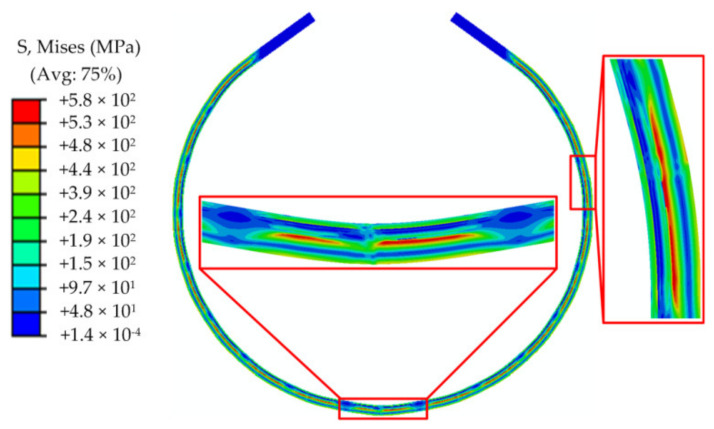
The Mises stress contour of results of the 2D model.

Figure 20 presents the simulated result of the 2D FE model. Comparing Figure 20 with Figure 9 are veals that the Mises stress distributions of the 2D and 3D models are almost the same in the direction of the plate thickness, and there is little difference in the magnitude of the stress. However, the 2D model does not show stress distribution in other directions of the plate, such as the upper surface of the plate, i.e., the inner surface of the pipe, which is directly in contact with the corrosive medium during service. The stress state of the inner surface is a key point in the study of the pipe stress, and so, a 2D model cannot replace a 3D model. If only the thickness direction of the stress distribution is of concern, then a 2D model can be employed because it is easier to converge than a 3D model and the calculation time is much shorter than that of the 3D model. In addition, because comparatively much smaller meshes can be divided in a 2D model, it is more suitable to study the local stress distribution. If more comprehensive results are desired, a 3D model is recommended.

**Figure 21:**
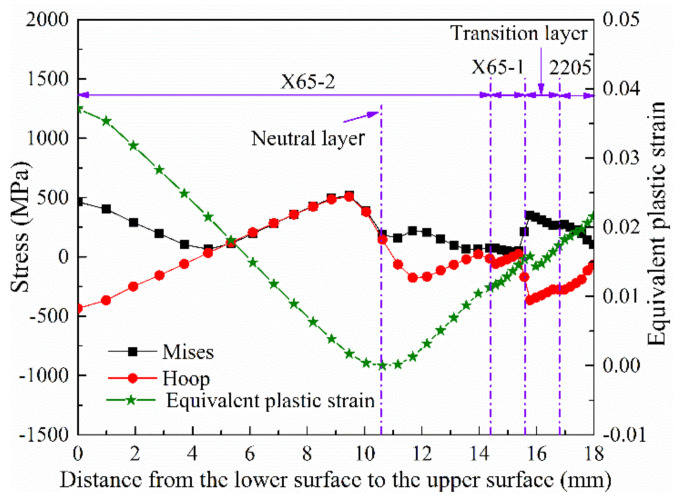
Stress and PEEQ distribution in the plate thickness direction after unloading.

To study the stress distribution at the interface of 2205 and X65, a path from the lower surface to the upper surface along the thickness direction, which is located at the center of the pressing position of the die, was selected. The stresses and strain distributions of the JCO forming after unloading were studied. The results are displayed in Figure 21. From the distribution of the equivalent plastic strain, it can be inferred that the deformation of the lower surface is the largest, followed by that of the upper surface. The equivalent plastic strain is 0 at 10.5 mm from the lower surface. This is also the neutral layer of the plate bending forming. The neutral layer of the bimetallic plate bending is closer to the 2205 layer, which has greater strength, whereas the neutral layer of a single metal plate bending is generally located in the middle of the thickness direction of the plate. The maximum Mises stress of the whole plate after the unloading is located 9.5 mm from the lower surface. After unloading, the hoop stress presents a compressive stress on the lower surface. With the increase in the distance from the lower surface to the upper surface, the compressive stress gradually decreases to 0 at a 4 mm location and then turns into tensile stress, which then increases. The tensile hoop stress reaches the maximum at a 9.5-mm location, then rapidly decreases to compressive stress, and, finally, the hoop stress also exhibits a compressive stress distribution on the upper surface. After unloading, the residual Mises stress distribution on the neutral layer is about 180 MPa, and the hoop stress is about 140 MPa. It can also be seen from Figure 21 that the stress changes abruptly at the interfaces of X65-2 and X65-1, X65-1 and the transition layer, and the transition layer and 2205, whereas the equivalent plastic strain does not change remarkably at the interfaces of the different materials.

## 4. Conclusions

In this study, 3D finite element models are established to study the pre-bending and JCO forming process of a 2205/X65 bimetallic pipe, and the simulation results are compared to the corresponding DIC experimental results. The appropriate shape of the pipe billet is obtained, and the stress evolution and distribution are analyzed. Furthermore, the influences of the plate sizes and the processing parameter on the residual stresses are numerically investigated. Finally, the interfacial stress distributions of different materials are analyzed by a 2D model, which has fine grids, and the simulation results of the 2D and 3D models are compared. Moreover, the free bending characteristics of bimetallic plates are pointed out. The following conclusions can be drawn:

1. The results of the finite element analysis are in good agreement with those of the DIC experiments.
2. The stress and PEEQ distributions of the formed pipe are segmented. The numbers of these segments are the same as the pressing times, and the stresses of each segment are mainly caused by the downward–upward pressing process of the die. The formed pipe billet is composed of alternate circular and straight segments. The opening width of the pipe billet exhibits a linear relationship with the press amount of the JCO upper die, and the appropriate press amount can be obtained by the finite element model, which is confirmed by experiment.
3. After the downward pressing process of the die, the stress of the pipe reaches maximum. The equivalent stress of the upper 2205 stainless steel is larger than that of the lower X65 pipe steel, which is related to the larger working-hardening of 2205 than that of X65 during the explosive welding. With the upward pressing of the die, a large elastic-recovery deformation occurs on the plate, and the stress appears to be significantly reduced. As the plate moves forward, the Mises stress and axial stress change little. The maximum shear stress of the pipe in the JCO forming process is much less than the shear strength of the plate, which indicates that the 2205 and X65 interface is well bonded after the JCO forming process.
4. The maximum hoop stress and the maximum tensile axial stress on the upper surface increase with increasing plate strength. With the increase in the plate thickness, the maximum tensile axial stress on the upper surface after the JCO forming increases linearly, whereas the hoop stress increases first and then tends to be stable. With the increase in the span, the maximum tensile axial stress on the upper surface of plate reduces significantly, whereas the hoop stress decreases first and then remains unchanged.
5. Comparing the results of the 2D and 3D models reveals that the result of the 2D model can only present the stress distribution in the plate thickness direction, and the simulated results in this direction are consistent with those of the 3D model. The neutral layer is closer to the 2205 layer, which has a higher yield strength compared to X65 layer. After unloading, maximum residual Mises stress distribution is found in the middle of the pipe thickness direction, and the hoop stress is compressive on both the upper and lower surfaces.
6. A large gradient transition of the stress is present at the interfaces of different materials in bimetallic pipe after JCO process.

## Figures and Tables

**Table 1:** Material properties of plate metal.

	E/GPa	μ	ρ/tonne/mm^3^
**X65**	210	0.3	7.85 × 10^−9^
**2205**	210	0.3	7.80 × 10^−9^

**Table 2:** Chemical composition of X65 pipe steel and 2205 duplex stainless steel (wt %).

	C	Si	Cr	Mn	Ni	Cu	Mo	V	P	S	Fe
X65	0.053	0.33	0.07	1.18	0.16	0.14	0.06	0.031	0.016	≤0.005	Balance
2205	0.021	0.56	22.59	1.13	5.29	-	3.45	-	0.018	0.001	Balance

**Table 3:** DIC and FEM results of the springback and bending angle.

Press Amount/mm	Springback/mm			Bending Angle/°		
	DIC	FEM	Relative Error %	DIC	FEM	Relative Error %
20	1.72	1.65	4.1	26.83	28.1	4.5
21	1.73	1.66	4.0	28.3	29.6	4.4
22	1.74	1.67	4.0	29.69	31.1	4.5

**Table 4:** Yield strength of the simulated plates.

Plate	Layer	Yield Strength/MPa
Plate 1	X65	508
	2205	530
Plate 2	X65	534
	2205	774
Plate 3	X65	560
	2205	1018

## References

[1] Yingsamphancharoen T., Srisuwan N., Rodchanarowan A. (2016). The electrochemical investigation of the corrosion rates of welded pipe ASTM A106 grade B. Metals.

[2] Liu Z.Y., Dong C.F., Li X.G. (2009). Stress corrosion cracking of 2205 duplex stainless steel in H_2_S–CO_2_ environment. J. Mater. Sci..

[3] Liu X., Zhang H., Wang B., Xia M., Wu K., Zheng Q., Han Y. (2018). Local Buckling Behavior and Plastic Deformation Capacity of High-Strength Pipe at Strike-Slip Fault Crossing. Metals.

[4] Wang X., Ye J.D., Ma X., Tian Q.Q., Li X., Bao Y. (2014). Finite element simulation analysis of multi-stands three-roller cold rolling process for double metal composite seamless steel tube. Appl. Mech. Mater..

[5] Yang X.Q. (2008). Current Situation, Existing Problems and Development Prospect of Domestic Steel Tube Industry (Part I). Steel Pipe.

[6] Li Y.F., Sun Q. (2004). Study and Application of JCOE LSAW Pipe Production Line. Welded Pipe Tube.

[7] Yanfeng L. (2009). R&D of Φ1219 mm X80 LSAW Linepipe for No.2 West-East Gas Transmission Pipeline Project. Steel Pipe.

[8] Zheng R., Li T.J. (2003). Production and Development of China’s Pipeline Steel. Heavy Mach. Technol..

[9] Barnes P., Hejazi R., Karrech A. (2018). Instability of mechanically lined pipelines under large deformation. Finite Elem. Anal. Des..

[10] Li R., Eyckens P., Gawad J., Poucke M., Cooreman S., Bael A. (2017). Advanced plasticity modeling for ultra-low-cycle-fatigue simulation of steel pipe. Metals.

[11] Kim N., Kang B., Lee S. (2003). Prediction and design of edge shape of initial strip for thick tube roll forming using finite element method. J. Mater. Process. Technol..

[12] Jiang J., Li D., Peng Y., Li J. (2009). Research on strip deformation in the cage roll-forming process of ERW round pipes. J. Mater. Process. Technol..

[13] Ren Q., Zou T., Li D., Tang D., Peng Y. (2015). Numerical study on the X80 UoePipe forming process. J. Mater. Process. Technol..

[14] Gao Y., Li Q., Xiao L. Numerical Simulation of JCO/JCOE Pipe Forming. Proceedings of the World Congress on Computer Science and Information Engineering.

[15] Luo J., Xue Y., Chen K., Shang Y., Zhang C. (2017). Integrated simulation and experimental test of the residual stress field for large-sized straight welded pipe processed with JCOE Technology. Int. J. Steel Struct..

[16] Ren Y., Paradowska A., Eren E., Wang B. Challenges of Measuring Residual Stresses in Large Girth Welded Pipe Spools by Neutron Diffraction. Proceedings of the International Conference on Residual Stresses.

[17] Chen W., Boven G.V., Rogge R. (2007). The role of residual stress in neutral PH stress corrosion cracking of pipeline steels—Part II: Crack dormancy. Acta Mater..

[18] Hino R., Goto Y., Yoshida F. (2003). Springback of sheet metal laminates in draw-bending. J. Mater. Process. Technol..

[19] Ling Y.E., Lee H.P., Cheok H.P. (2005). Finite Element Nnalysis of Springback in L-bending of Sheet Metal. J. Mater. Process. Technol..

[20] Gao Y., Li Q., Fan L., Wang X. Applications of Finite Element Method in Large-diameter Longitudinal Welded Pipe. Proceedings of the International Conference on Measuring Technology and Mechatronics Automation.

[21] Zhang L.J., Pei Q., Zhang J.X., Bi Z.Y., Li P.C. (2014). Study on the microstructure and mechanical properties of explosive welded 2205/X65 bimetallic sheet. Mater. Des..

[22] Gou N.N., Zhang L.J., Zhang J.X. (2018). Increased quality and welding efficiency of laser butt welding of 2205/X65 bimetallic sheets with a lagging MIG arc. J. Mater. Process. Technol..

[23] Dong Z.Q., Zhang J.X. (2018). Three-dimensional finite element analysis of residual stresses in circumferential welds of 2205/X65 bimetallic pipe. Int. J. Adv. Manuf. Technol..

[24] Tsai W.T., Chen M.S. (2000). Stress corrosion cracking behavior of 2205 duplex stainless steel in concentrated NaCl solution. Corros. Sci..

[25] Fukuda N., Hagiwara N., Masuda T. (2005). Effect of prestrain on tensile and fracture toughness properties of line pipes. J. Offshore Mech. Arct. Eng..

[26] Nixon M.E., Lebensohn R.A., Cazacu O., Liu C. (2010). Experimental and finite-element analysis of the anisotropic response of high-purity α-titanium in bending. Acta Mater..

